# Supplementary material for: Determinants of age-related decline in walking speed in older women
Source: PeerJ. 2023 Mar 7;11:e14728. doi: 10.7717/peerj.14728 (PMC10007973; doi:10.7717/peerj.14728)
Supplement: Supplemental Information 2 [file peerj-11-14728-s002.docx]

**CODEBOOK OF DATASET**

SSWS: spontaneous velocity

SSWMS: maximal velocity

WR: walking ratio

LRI: locomotor rehabilitation index

age group: 1 - 60-69 years; 2 - 70-79 years; 3 - 80-100 years

sex: 1- woman; 2 - man
